# The Importance of 14‐3‐3 Proteins in Colorectal Cancer Regulation: Mechanisms of Function and Clinical Opportunities

**DOI:** 10.1155/bmri/1090181

**Published:** 2026-06-13

**Authors:** Samaneh Dodge, Arman Soltani Moghadam, Mahmoud Khansari, Ahmad Mir Hosseini, Mona Torkaman Cheh, Shila Fallahpour, Yasaman Ghodsi Boushehri, Soheil Bolandi, Sepide Javankiani, Hossein Gharedaghi, Dejbakht Majid, Zahra Hasanabadi, Faeze Ahmadi Beni, Qumars Behfar

**Affiliations:** ^1^ School of Pharmacy, Tehran University of Medical Sciences, Tehran, Iran, tums.ac.ir; ^2^ School of Medicine, Tehran University of Medical Sciences, Tehran, Iran, tums.ac.ir; ^3^ Department of Medical Genetics, School of Medical Sciences, Tarbiat Modares University, Tehran, Iran, modares.ac.ir

**Keywords:** 14-3-3 proteins, colorectal cancer, protein interaction, tumor progression

## Abstract

Colorectal cancer (CRC) is the third most frequent cancer and one of the deadliest cancers, with a significant global disease burden. Despite progress in the diagnosis and treatment of CRC, the rate of mortality is still high. Identifying the molecular mechanisms contributing to CRC development and progression creates opportunities to provide better management for CRC. 14‐3‐3 proteins are extremely conserved phosphoserine/phosphothreonine‐binding proteins found in all eukaryotic cells. These proteins regulate a number of biological processes in the cells by binding to their client proteins. Thereby, they can contribute to the regulation of cell cycle progression, proliferation, invasion, and metastasis, highlighting their role in cancer development, progression, and treatment. The present article is aimed at reviewing the 14‐3‐3 proteins and their biological function, as well as their contribution to cancer regulation. In addition, it will collect previously performed studies investigating the role of 14‐3‐3 proteins in CRC pathogenesis, emphasizing their potential for diagnostic, prognostic, and therapeutic purposes, offering insights into novel intervention strategies and personalized medicine approaches for CRC.

## 1. Introduction

Colorectal cancer (CRC), a significant global health challenge, is reported as the third most frequent cancer and the second most lethal cancer worldwide. CRC was relatively rare in the 1950s, but its incidence has increased in western countries and now accounts for nearly 10% of cancer‐related mortality worldwide [1, 2].

CRC can arise from both environmental and genetic influences. Insufficient physical activity and obesity, inflammatory bowel disease, high‐fat diet, alcohol intake, and family history are reported as risk factors for CRC development [3]. Numerous symptoms facilitate the early detection of CRC. Skalitzky et al. performed a retrospective cohort study to describe the specific symptoms of patients suffering from CRC. Their findings showed that changes in bowel habits, rectal bleeding, and less common abdominal pain were the most frequent presenting symptoms, with many patients exhibiting at least one of these symptoms. In addition, fever, weight loss, fatigue, and early satiety or anorexia were also common. Furthermore, abdominal manifestations such as constipation, diarrhea, and distention were also observed. Back pain, rectal pain, anemia, urinary symptoms, obstruction, and pelvic pressure were found to be less common [4].

Traditional treatments used at present for CRC are surgery for the resectable tumors and chemotherapy combined with radiotherapy for unresectable tumors [5]. Nonetheless, surgery is ineffective for later stages, and chemotherapy and radiotherapy lack specificity. Additionally, these approaches may harm normal tissues, leading to secondary complications and unwanted outcomes. Moreover, patients often acquire resistance to chemotherapy, diminishing the efficiency of the treatment and enhancing the possibility of tumor recurrence [6]. These factors may explain why mortality in CRC remains high despite therapeutic advances. This highlights the need for other management approaches to overcome these issues. Progress in molecular biology and genomics has opened a new era of targeted treatment against cancer [5]. Understanding different cellular and molecular mechanisms underlying CRC development and progression allows us to identify novel diagnostic, prognostic, and therapeutic approaches [7].

14‐3‐3 proteins have received great attention in recent years. They bind to phosphorylated proteins, affecting their function and localization, regulating various biological processes. Thereby, they have been implicated in the development of different illnesses, such as heart abnormalities [8], metabolic disease [9], neurodegenerative disorders [10], and so on. 14‐3‐3 proteins are also known as important regulators of cancer through their contribution to several signaling pathways and biological events, including cell proliferation, growth, invasion, apoptosis, and resistance to therapy, functioning as oncogenes or tumor suppressors [11]. Numerous studies have evaluated the different roles of the 14‐3‐3 protein family in a variety of cancers. This review is aimed at providing a comprehensive overview of the role of 14‐3‐3 proteins in CRC, highlighting their contribution to critical signaling pathways, clarifying their diagnostic and prognostic application, and underscoring their potential as innovative therapeutic targets.

## 2. 14‐3‐3 Proteins: Structure and Function

**Figure 1:**
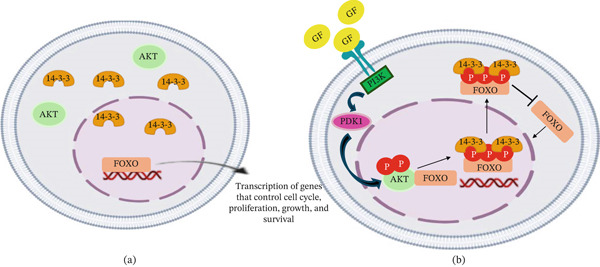
Schematic representation of regulation of FOXO activity by 14‐3‐3 proteins. (a) In resting cells, FOXO binds to DNA through its DNA‐binding domain, promoting the expression of its responding genes that contribute to the regulation of cell cycle, proliferation, and cell survival. (b) When the cell receives the signals stimulating PI3K/AKT signaling, AKT gets activated and causes phosphorylation of FOXO protein, providing two binding sites for 14‐3‐3, which leads to 14‐3‐3 physically binding to the DNA‐binding domains of FOXO, masking the DNA‐binding surface that prevents FOXO from binding to the target DNA and making it leave the nucleus, leading to inhibition of the expression of its responding genes. In addition, 14‐3‐3 masks the nuclear localization sequence of FOXO protein, preventing its return to the nucleus.

The 14‐3‐3 protein family is an extremely conserved group of proteins found in all eukaryotic cells. 14‐3‐3 proteins were discovered in the 1960s by Moore and Perez (or Moore and Pretz) during the analysis of bovine brain proteins. The unique name 14‐3‐3 is rooted in their elution pattern in gel‐filtration fraction and their position on a starch gel electrophoresis [12]. Following their discovery, they remained largely overlooked for several decades. However, they resurfaced in the 1990s due to their determined roles, such as regulation of Raf‐1 kinase, tyrosine and tryptophan hydroxylase, and protein kinase C. After that, the number of proteins that interact with and are regulated by the 14‐3‐3 family grew, with over 1200 14‐3‐3‐binding proteins identified in mammals [13]. The 14‐3‐3 family encompasses seven types of proteins represented by the Greek letters *σ*, *ε*, *ζ*, *γ*, *β*, *θ*, and *η*, encoded by genes *SFN* (Stratifin), *YWHAE*, *YWHAZ*, *YWHAG*, *YWHAB*, *YWHAQ*, and *YWHAH*, respectively. Discovering the structure of 14‐3‐3 proteins revealed that these proteins form homodimers or heterodimers, and each monomer is made up of nine antiparallel *α*‐helices that create a conserved amphipathic groove. This groove serves as the primary binding site for protein interactions, usually identifying phosphorylated serine/threonine‐containing motifs. This dimerized form and conserved binding construction enable 14‐3‐3 proteins to interact with various target proteins simultaneously, acting as molecular scaffolds in diverse signaling pathways [13, 14]. 14‐3‐3 protein–protein interactions represent a specific phosphorylation context, and three conserved binding motifs are reported as pS/TX COOHT, RXXpS/TXP, and RX (F/Y) XpS/TXP [15]. Thereby, they can affect the stability, localization, or activity of their binding partners. Indeed, the exact effect of each 14‐3‐3 protein on its binding proteins varies depending on the unique client protein. However, the ability of 14‐3‐3 proteins to interact with other proteins and regulate them allows these proteins to contribute to the modulation of various signaling pathways and cellular processes [16]. For example, according to Masone et al., binding of 14‐3‐3*ζ* to arylalkylamine N‐acetyltransferase (AANAT), an enzyme involved in the biosynthesis of melatonin, enhances the affinity of the enzyme to its substrates, causing the stimulation of melatonin production even at low substrate amounts [17]. As another example of contribution of 14‐3‐3 proteins to regulation of biological processes, binding of 14‐3‐3 proteins to a location near the nuclear localization signal (NLS) of phosphorylated forkhead box O transcription factors (FOXO), a transcription factor involved in DNA damage repair, cell survival, and stress resistance, could disturb the nuclear localization function of NLS, causing FOXO to leave the nucleus, preventing its transcriptional activity (Figure 1). Obsilova et al. supported this function of 14‐3‐3 proteins by reporting direct interaction of 14‐3‐3*ζ* with NLS of FOXO4 [18, 19]. Cell division cyclin 25 (Cdc25), playing a role in G2/M transition of the cell cycle, also has a binding site for 14‐3‐3 proteins close to the NLS. Thereby, the interaction of 14‐3‐3 proteins with phosphorylated Cdc25 causes its translocation to the cytoplasm, preventing its ability to promote the G2‐M transition into mitosis. Uchida et al. demonstrated that formation of the 14‐3‐3*ε*/Cdc25C complex interferes with NLS activity, thereby allowing the N‐terminal nuclear export signal (NES) to predominate and promote Cdc25 export from the nucleus to the cytoplasm [20]. By attaching to their partners and preventing phosphorylated motifs from being dephosphorylated, 14‐3‐3 proteins can also control cellular functions. For example, death‐associated protein kinase 2 (DAPK2) is a serine/threonine kinase, playing a role in several cellular functions, including programmed cell death, autophagy, and granulocyte development. It functions as a tumor suppressor in various types of cancer by maintaining mitochondrial health and triggering the NF‐*κ*B signaling pathway. The activity of DAPK2 is regulated through multiple mechanisms, including autophosphorylation and formation of dimers. According to a report by Horvath et al., 14‐3‐3*γ* binds to the phosphorylated site (Ser318) of DAPK2 and protects it from dephosphorylation, removing the dimer structure, thereby inhibiting its activation [21]. Furthermore, 14‐3‐3 proteins can exert their effects by attaching to a protein and blocking its interaction with other proteins or DNA. Caspase 2 is the most conserved member of the caspase family, required for the correct initiation of apoptosis in response to cell death signals. Caspase 2 becomes activated via dimerization and self‐cleavage (autoproteolysis). Kalabova et al. represented that binding 14‐3‐3 proteins to the Caspase 2 masks its p12 domain, which contributes to dimerization. Therefore, 14‐3‐3 proteins suppress pro‐Caspase 2 activation by preventing its dimerization interface [22]. Furthermore, Silhan et al. indicated that 14‐3‐3*ζ* protein binds to DNA‐binding domains (DBD) of FOXO4, potentially concealing the DNA‐binding surface and prohibiting FOXO4 from binding to the target DNA (Figure 1) [23]. Furthermore, 14‐3‐3 proteins can act as a scaffold, maintaining two proteins near each other. Activation of BRAF, a signaling molecule crucial for the RAS/MAPK pathway, is mediated by its dimerization. Where the stimulating factors trigger the RAS/MAPK pathway, activated Ras‐GTP binds to the Ras‐binding domain (RBD) of BRAF, causing the c‐terminus to be phosphorylated at specific sites, such as pSer729. This phosphorylation then allows the 14‐3‐3 dimer to bind and bridge BRAF monomers together, inducing the formation of an active BRAF dimer [24].

Beyond the activities of 14‐3‐3 proteins in the cell, it is necessary to point out that although 14‐3‐3 proteins are primarily localized within the cell, they can be found in the extracellular environment, contributing to normal cellular activities and specific disorders. These proteins bind to aminopeptidase N (APN), a cell‐surface receptor, and promote matrix metalloproteinase (MMP) activity and tissue remodeling. They can also have proinflammatory and immunoregulatory activities [25].

Therefore, 14‐3‐3 proteins could interact with a range of proteins, including receptors, transcription factors, signaling hub proteins, and so on, contributing to various cellular processes such as signal transduction, cell cycle control, metabolism, apoptosis, and protein trafficking.

## 3. 14‐3‐3 Proteins in Cancer Regulation

In previous sections, we discussed the various actions of 14‐3‐3 proteins and their importance to biological processes. As a result, it is not surprising that these magnificent proteins have an undeniable role in cancer formation. There is a growing number of studies evaluating multiple roles of 14‐3‐3 proteins in various cancers [26–29]. In this part, we describe some original articles, suggesting various roles for 14‐3‐3 in the pathogenesis of cancer through the regulation of different biological events.

### 3.1. 14‐3‐3 Proteins Regulate Cell Cycle Progression and Cell Proliferation

Uncontrolled cell growth is an initial stage in the development of tumors, and the first evidence supporting the contribution of 14‐3‐3 proteins in cell proliferation emerged in 1994 by Morrison et al., as they uncovered that these proteins interact with several proteins having oncogenic potential, such as members of the Raf kinase family [30]. Since then, the number of investigations supporting the role of 14‐3‐3 proteins in cancer initiation and progression through the regulation of cell cycle progression and cell proliferation has increased. Wu et al. indicated that knockdown of 14‐3‐3*β*, an upregulated protein in osteosarcoma, caused decreased cell proliferation and cell cycle progression in MG63 cells. Their findings also suggested that 14‐3‐3*β* exerts its oncogenic roles through regulating *β*‐catenin expression, thereby promoting the Wnt/*β*‐catenin signaling pathway [31]. The apoptosis stimulating protein of p53 2 (ASPP2) is recognized as an antitumor protein that is downregulated in cancer. A study conducted by Yang et al. represented the contribution of 14‐3‐3 proteins in the underlying mechanism by which ASPP2 represses cell proliferation. 14‐3‐3 interacts with the phosphorylated kinase domain of BRAF, stimulating cell proliferation by attenuating the MEK/ERK signaling. ASPP2 binds to 14‐3‐3, preventing its interaction with BRAF and thereby stimulating BRAF/MEK/ERK activation, which leads to the suppression of cell proliferation in hepatocellular carcinoma (HCC) [32]. Miz1 is a transcription factor essential for initiating cell‐cycle arrest responding to DNA damage. The protein 14‐3‐3*η* represses its activity by directly binding to it, thereby partially reducing Miz1‐induced cessation of cells in the G1 phase. Additionally, 14‐3‐3*η* and Miz1 regulate a number of genes contributing to cell proliferation or upregulated genes in cancers [33]. Polo‐like kinase 1 (PLK1) exerts a crucial role in cell division, regulating several important steps in mitosis and meiosis. Therefore, the regulation of PLK1 function has a significant importance for its role in spindle assembly, maturation of centrosome, microtubule and kinetochore binding, and cytokinesis. According to Kasahara et al., Phosphorylation of PLK1 at Ser99 by AKT generates a binding site for 14‐3‐3*γ*, and this binding enhances enzymatic activity of PLK1, which is essential for mitosis regulation. Downregulation of 14‐3‐3*γ*, PI3K, or AKT leads to a cell‐cycle arrest in prometaphase or metaphase [34].

The described examples support the idea that 14‐3‐3 proteins could regulate cancer development and progression by impacting cell cycle progression.

### 3.2. 14‐3‐3 Proteins Regulate Apoptosis in Human Tumors

Beyond the role of 14‐3‐3 proteins in cell cycle control, these proteins are key regulators of apoptosis and survival pathways. 14‐3‐3*η* is reported to be involved in carcinogenesis by attenuating apoptosis of cancer cells. Immunofluorescence by Park et al. indicated the higher expression of 14‐3‐3*η* in the nucleus of glioblastoma cells compared with the cytoplasm. Knockdown of 14‐3‐3*η* enhanced cell death by 30%, which occurred through both caspase‐dependent and caspase‐independent manners and made the cells more susceptible to microtubule‐inhibiting treatments [35]. BRCA1‐associated protein 1 (BAP1) is a nuclear deubiquitinating enzyme contributing to cell death. Wang et al. have demonstrated that in response to DNA damage, 14‐3‐3*τ* binds to E2F1 that has been phosphorylated by ATM. This interaction increases the stability of E2F1 and promotes the activation of its proapoptotic target genes, such as p73, caspases, and Apaf1, highlighting the significant role of 14‐3‐3*τ* in apoptosis triggered by DNA damage [36]. Consistently, Sim et al. revealed that the proapoptotic activity of BAP1 is caused by its interaction with 14‐3‐3 proteins, stimulating cell death signaling in neuroblastoma [37]. Immunohistochemical examination of skin cancer revealed that elevated levels of 14‐3‐3*ε* contribute to the development and progression of the disease. Holmes et al. showed that 14‐3‐3*ε* is upregulated in the cytoplasm of SCC cells and represses apoptosis through promoting prosurvival signaling by activating Survivin, P‐Akt (Ser473), and P‐BAD (S136), facilitating skin carcinogenesis. They showed that 14‐3‐3*ε* relocalized and sequestered Survivin into the cytoplasm, making the apoptotic effects of Survivin. Knockdown of 14‐3‐3*ε* was followed by a 65% increase in apoptosis and reduced phosphorylation of Bad and AKT proteins. Additionally, targeted inhibition of 14‐3‐3*ε* in mice led to a 75% reduction in skin tumor formation [38].

### 3.3. 14‐3‐3 Proteins Regulate Cell Migration, Invasion, and Metastasis in Cancer

Beyond proliferation and survival, 14‐3‐3 proteins also play significant roles in cell migration and metastasis. PIM1 is a serine/threonine kinase upregulated in prostate cancer that mediates the overexpression of genes playing a role in cell migration and invasion. Ruff et al. uncovered that this effect of PIM1 is mediated by phosphorylation of 14‐3‐3 *ζ* and the androgen receptor (AR), allowing them to bind to each other and recruit other AR coregulators, including hnRNPK and TRIM28, finally causing overexpression of genes involved in migration [39]. In addition, an investigation performed by Xiao et al. reported that 14‐3‐3*τ* stimulates breast cancer invasion and metastasis via attaching to RhoGDI*α* and inhibiting it, which is a negative regulator of Rho GTPases and a metastasis inhibitor. Overexpression of 14‐3‐3*τ* in MCF7 breast cancer cells, which have endogenous low levels of this protein, leads to enhanced motility, reduced cell adhesion, and facilitated metastasis in mammary fat pad xenografts. Conversely, silencing 14‐3‐3*τ* in both MCF7 cells and the highly invasive MDA‐MB231 cell line suppresses Rho GTPase activation, thereby inhibiting cell invasion and migration. Furthermore, elevated 14‐3‐3*τ* expression in human breast tumors is correlated with ROCK activation (a downstream effector of Rho GTPase), an increased metastatic potential, and poorer patient survival outcomes. Considering these observations, the study suggested 14‐3‐3*τ* as a promising therapeutic target for preventing breast cancer progression and metastasis [40]. In addition, a study exploring the contribution of 14‐3‐3*β* in HCC cells represented that 14‐3‐3*β* stimulates migration capability and invasiveness in HCC cells via the upregulation of MMP 2 and 9 through the PI3K/Akt/NF‐*κ*B signaling pathway. In addition, a correlation was found between the enhancement of 14‐3‐3*β* and p‐Akt in the primary tumors of HCC patients, suggesting that combination detection of both 14‐3‐3*β* and p‐Akt predicts more accurately postoperative outcomes of HCC patients [41].

The epithelial‐mesenchymal transition (EMT) is a biological process characterized by decreased cell–cell adhesion and cell polarity of epithelial cells, accompanied by enhanced migration ability and invasiveness to become mesenchymal cells [42]. Increasing evidence has supported the contribution of 14‐3‐3 proteins in the regulation of EMT. According to a study performed by Liu et al., 14‐3‐3*ε* stimulates migration of HCC cells by EMT with an elevation of SNAIL, N‐cadherin, vimentin, and ZEB1, and a reduction of E‐cadherin. Knockdown of 14‐3‐3*ε* led to reduced cell migration and EMT [43]. In addition, it is reported that 14‐3‐3*γ* contributes to the EMT ability of nonsmall cell lung cancer cells. Inhibition of 14‐3‐3*γ* by Raungrut et al. repressed EMT, characterized by the reduced expression of EMT markers including E‐cadherin, N‐cadherin, and vimentin, and reduced migration (by 39%) and invasion (by 59%) of A549 cells, accompanied by the reduction of expression of MMP‐2 and MMP‐9 [44].

The described investigations highlight the undeniable role of 14‐3‐3 proteins in the migration, invasion, and metastasis ability of cancer cells, suggesting these proteins as valuable biomarkers for patient outcome prognosis and reasonable targets for cancer therapy.

### 3.4. 14‐3‐3 Proteins Could Affect Therapy Resistance in Cancer Treatment

The 14‐3‐3 proteins contribute significantly to therapy resistance in cancer by modulating key signaling pathways involved in DNA damage response, apoptosis, and cell survival. For example, ectopic expression of 14‐3‐3*σ* by Neupane et al. in the pancreatic cancer cell line Panc‐1 enhanced the survival rate of the cells in response to cisplatin treatment, while silencing 14‐3‐3*σ* in T3M4 cells increased apoptosis following treatment with cisplatin [45]. However, the majority of studies reporting the role of 14‐3‐3 proteins in chemoresistance and poor prognosis focus on the 14‐3‐3*ζ* gene, located on 8q22.3, a commonly duplicated region in cancer. Upregulation of 14‐3‐3*ζ* has been accompanied by poor patient outcomes in cancers, including lung, head and neck, breast, multiple myeloma, and glioblastoma. The rates of metastatic recurrence have been successfully decreased by adjuvant chemotherapy after breast cancer surgery. Nonetheless, many women still experience cancer recurrence at distant sites despite receiving such treatment. Identifying the key genes that influence tumor response to chemotherapy agents remains challenging but is essential for enhancing treatment outcomes. According to Li et al., overexpression of 14‐3‐3*ζ* caused by amplification of 8q22 is correlated with early disease recurrence despite adjuvant chemotherapy with anthracycline. The study reported that silencing this antiapoptotic protein increased the sensitivity of tumor cells to anthracyclines [46]. The binding of 14‐3‐3*ζ* to the Ser28‐phosphorylated form of histone H3 (H3S28ph) preserves the phosphorylation state of histone H3, which contributes to malignant transformation. Thereby, 14‐3‐3*ζ* is reported as a cause of treatment resistance and a regulator of the expression of genes contributing to EMT and stemness, such as *SRSF1*, *E2F2*, and *ID1*. Recently conducted research by Saito et al. found that benzaldehyde (BA) suppresses several pathways stimulated in cancer by preventing the interaction of 14‐3‐3*ζ* with its partners. However, the study indicated that lung cancer cells (A549 cell line) without endogenous 14‐3‐3*ζ* expression were less sensitive to the antitumor effects of BA than were control cells, suggesting the idea that 14‐3‐3*ζ* is necessary for the tumor‐inhibitory role of BA [47].

In addition, Qi et al., exploring the function of 14‐3‐3*γ* in lung cancer, found that enhanced levels of 14‐3‐3*γ* were linked to unregulated DNA replication, abnormal increases in DNA content, chromosome duplication, and increased resistance to mitotic inhibitors [48].

Furthermore, succinylation of 14–3–3*θ* by CPT1A, a succinyltransferase markedly overexpressed in extranodal natural killer/T‐cell lymphoma, nasal type (ENKTL‐NT), causes the stability of the protein, followed by increased cell survival and paclitaxel resistance [49].

These examples underscore that aberrant expression of 14‐3‐3 isoforms can rewire apoptotic and survival networks, allowing malignant cells to withstand chemotherapy, radiotherapy, and targeted agents. Therefore, 14‐3‐3 proteins represent potential biomarkers of therapy resistance and promising therapeutic targets in oncology.

Collectively, 14‐3‐3 proteins serve as multifunctional hubs in cancer regulation, integrating signals from proliferative, apoptotic, and migratory pathways. Their context‐dependent functions—as tumor suppressors or oncogenic drivers—underscore the intricacy of targeting this family in cancer treatment, while also establishing them as potential biomarkers and therapeutic candidates.

## 4. 14‐3‐3 Proteins′ Contribution to CRC

Various studies have explored the function of 14‐3‐3 proteins in CRC development and progression (Table 1).

**Table 1:** Expression patterns, molecular interactions, and functional roles of 14‐3‐3 protein isoforms in colorectal cancer.

14‐3‐3 isoform	Expression	Binding protein	Function	Clinical application	Role	Ref
14‐3‐3*σ*	Upregulated	Not mentioned (N)	Reduction of survival time	Therapeutic	Oncogene	[50]
14‐3‐3*σ*	N	N	Induction of G2 arrest in response to DNA‐damaging	N	Tumor suppressor	[51]
14‐3‐3*σ*	Upregulated	N	Stimulation of tumorigenesis, cell proliferation, and progression	Therapeutic	Oncogene	[52]
14‐3‐3*σ*	Downregulated	SNAIL, c‐JUN, YAP1, and FOXO1	Inhibition of proliferation, metastasis, and EMT	Prognostic	Tumor suppressor	[53]
14‐3‐3*σ*	Downregulated	HIF‐1*α*	Inhibiting hypoxia‐induced CRC metastasis and angiogenesis	Therapeutic	Tumor suppressor	[54]
14‐3‐3*σ*	Downregulated	AKT	Inhibition of migration and invasion	Therapeutic	Tumor suppressor	[55]
14‐3‐3*ε*	Upregulated	Bad	Inhibition of apoptosis	Preventive, therapeutic	Oncogene	[56]
14‐3‐3*ε*	Downregulated	hnRNP C	Suppression of proliferation, induction of cell‐cycle arrest at the S phase, and autophagy	N	Tumor suppressor	[57]
14‐3‐3*ε*	N	N	Negative regulation of lymph node metastasis	Prognostic	Tumor suppressor	[58]
14‐3‐3*ζ*	Upregulated	AMPK	Stimulation of cell proliferation and tumor growth, inhibition of apoptosis	Therapeutic and prognostic	Oncogene	[59]
14‐3‐3*ζ*	Upregulated	TRIP13	Mediates G2‐M transition and EMT	Therapeutic	Oncogene	[60]
14‐3‐3*ζ*	Upregulated	FOXO3	Stimulation of proliferation and cell survival	Therapeutic	Oncogene	[61]
14‐3‐3*β*	Upregulated	N	Associated with microsatellite instability	Prognostic	Oncogene	[62]
14‐3‐3*β*	Upregulated	PIK3R2	Stimulation of proliferation, inhibition of cell‐cycle arrest in G0/G1, and apoptosis	Therapeutic	Oncogene	[63]
14‐3‐3*γ*	Upregulated	*β*‐catenin		Therapeutic and prognostic	Oncogene	[64]
14‐3‐3*η*	Upregulated	N	Causing immunotherapy resistance	Therapeutic	Oncogene	[65]

For example, Young et al. performed real‐time PCR to assess if there are any differences between the expressions of 14‐3‐3 isoforms in CRC tissues compared with nontumor tissues. Their observations showed that the expression of 14‐3‐3 *δ*, *η*, and *ζ* was considerably lower in adenocarcinoma samples compared with adjacent normal tissues. Analyzing the datasets and comparing the normal and cancerous samples obtained from The Cancer Genome Atlas (TCGA) confirmed their qRT‐PCR findings. However, they found no correlation between expression alterations and the patient′s gender, age, or race. Interestingly, the level of expression of 14‐3‐3 proteins could vary in patients with different stages of CRC. TCGA dataset analysis revealed that the most significant fluctuation was observed for 14‐3‐3*β*, downregulated in early‐stage tumors but upregulated in late‐stage tumors. Additionally, 14‐3‐3*γ*, a tumor‐promoting 14‐3‐3 protein, showed enhanced expression in more advanced tumors. In contrast, advanced cancers showed lower expression levels of 14‐3‐3*δ*, a putative tumor suppressor, compared with early‐stage malignancies. Moreover, evaluating the methylation status of the promoter represented a wide range of methylation at the 14‐3‐3*η* promoter, suggesting this member of the 14‐3‐3 family as a tumor suppressor [66].

Although these findings highlight the differential expression of 14‐3‐3 isoforms in CRC, their association with patient survival and prognosis has only been partially explored. For example, loss of nuclear expression of 14‐3‐3*ε* has been significantly associated with poor overall survival and has been identified as an independent prognostic factor in CRC patients [58]. Similarly, 14‐3‐3*σ* has been reported to be associated with patient survival, with several studies indicating that its altered expression correlates with poor prognosis in CRC [50]. However, the prognostic implications of 14‐3‐3 proteins appear to be isoform‐ and context‐dependent, as some studies suggest dual or even opposing roles in tumor progression. Notably, earlier expression studies, including that of Young et al., did not directly assess survival outcomes, highlighting a gap in the literature [66]. Therefore, further large‐scale and systematic analyses are required to clarify the prognostic value of different 14‐3‐3 isoforms in CRC. Table 2 represents the association of 14‐3‐3 isoforms with patient survival and prognosis in CRC.

**Table 2:** Association of 14‐3‐3 isoforms with patient survival and prognosis in colorectal cancer.

Isoform	Expression pattern in CRC	Survival outcome (OS/DFS)	Prognostic value	Type of evidence	Reference
14‐3‐3*ε*	Reduced (especially nuclear)	Decreased overall survival	Independent prognostic factor	Clinical cohort (IHC)	[58]
14‐3‐3*σ*	Downregulated	Poor prognosis (context‐dependent)	Context‐dependent prognostic marker	Clinical observation	[54]
14‐3‐3*β*	Stage‐dependent	Not well established	Unclear	TCGA/expression study	[66]
14‐3‐3*γ*	Upregulated	Limited evidence (possible poor survival)	Suggested prognostic relevance	Expression‐based	[64]
14‐3‐3*ζ*	Upregulated	Not clearly defined	Potential prognostic marker	Experimental + limited clinical	[59]
14‐3‐3*η*	Upregulated	Unknown	Not established	Limited evidence	[65]

Abbreviations: DFS, disease‐free survival; OS, overall survival.

14‐3‐3*σ*, an acidic dimeric protein, has been reported as a tumor suppressor that is downregulated in several cancers. However, its enhanced expression is suggested to be correlated with poor prognosis and a cause for resistance to anticancer therapies and radiation causing damage [67]. Therefore, considering the different roles of 14‐3‐3 in various cancers as an oncogene or tumor suppressor, some studies evaluated 14‐3‐3*σ* expression and function in CRC. For example, Perathoner et al. employed western blot to check the expression of 14‐3‐3*σ* in nine CRC cell lines and compare its expression between eight CRC tissues and adjacent normal tissues. Their findings showed a significant overexpression of 14‐3‐3*σ* in four of eight cell lines and in six of eight colorectal carcinoma tissues. In addition, immunohistochemistry of 121 CRC patients represented upregulation of 14‐3‐3*σ* in 38.8% of CRC samples. Their observations revealed a correlation between highly positive immunoreactivity and tumor differentiation (*p* < 0.001) as well as pT stage (*p* < 0.003). Furthermore, Kaplan–Meier analysis showed a significant association between the overexpression of 14‐3‐3*σ* and reduced survival time. Finally, the authors suggested 14‐3‐3*σ* as a logical therapeutic target in CRC [50].

**Figure 2:**
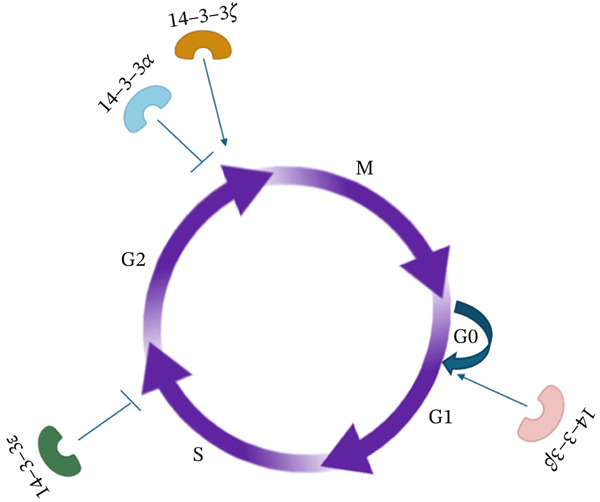
Regulation of cell cycle progression by 14‐3‐3 proteins in CRC. 14‐3‐3*σ* induces G2 arrest, preventing cell cycle progression from G2 to mitosis, and inhibits cell division and proliferation; 14‐3‐3*ε* causes cell‐cycle arrest at the S phase, inhibiting proliferation of CRC cells; 14‐3‐3*ζ* exerts an oncogenic role by mediating G2/M transition; and 14‐3‐3*β* inhibits cell‐cycle arrest in G0/G1, facilitating cell‐cycle progression and proliferation.

14‐3‐3 proteins could contribute to CRC by regulating the cell cycle (Figure 2). Exposure of CRC cells to ionizing radiation causes cell‐cycle arrest in both G1 and G2 phases. Although G1 arrest was known to be induced by p53‐mediated induction of the cyclin‐dependent kinase inhibitor p21WAF1/CIP1/SDI1, scientists conducted a quantitative analysis of gene expression patterns to investigate the mechanism underlying G2 arrest. Their findings showed that gamma irradiation and other DNA‐damaging factors strongly induce 14‐3‐3*σ*, which is caused by a p53‐responsive region located 1.8 kb upstream of its transcription start point. Furthermore, they showed that exogenous induction of 14‐3‐3*σ* into cycling cells leads to a G2 arrest [51]. Findings from Tanaka et al. in gastric and CRCs indicated a possible p53‐independent regulatory pathway for 14‐3‐3*σ*. Using RT‐PCR and western blot analyses, they observed that 14‐3‐3*σ* was markedly upregulated in tumor tissues compared with adjacent normal samples. In CRC, strong cytoplasmic staining was associated with lymph node metastasis. Finally, they suggested that 14‐3‐3*σ* could stimulate tumorigenesis, cell proliferation, and progression of human gastrointestinal cancers [52].

**Figure 3:**
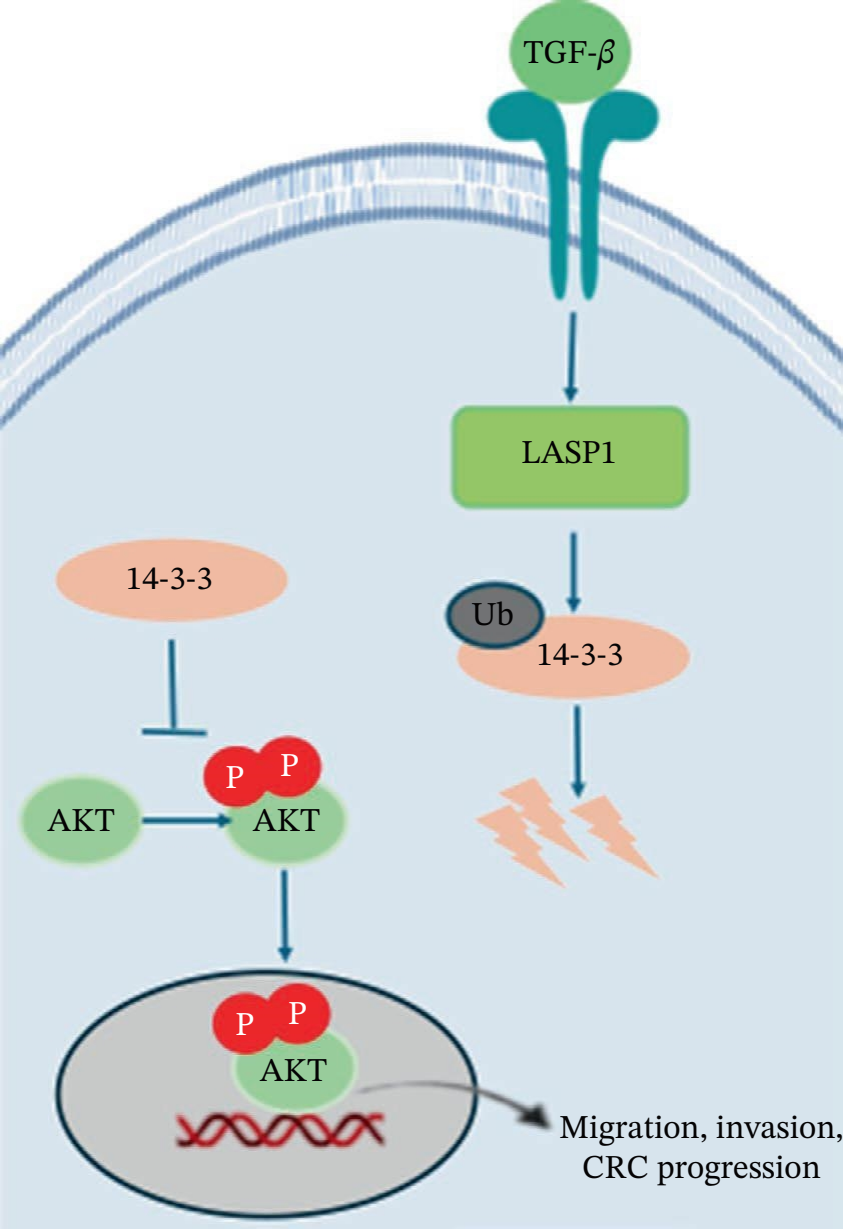
A schematic illustration of regulating the PI3K/AKT pathway by 14‐3‐3 *σ* in CRC. Phosphorylated AKT binds to DNA, causing expression of genes involved in cell migration, invasion, and CRC progression. 14‐3‐3*σ* interacts with AKT and inhibits its phosphorylation by AKT kinase, preventing its interaction with DNA. LASP1, a positive regulator of PI3K/AKT, causes ubiquitination of 14‐3‐3*σ*, leading to its degradation and suppressing its inhibitory effects on AKT activation.

Winter et al. found that 14‐3‐3*σ* levels were lower in CRC tissues, contradicting the findings of Perathoner et al. and Anaka et al. The knockout of 14‐3‐3*σ* in *APC*‐mutant mice led to a considerable increase in both the number and size of adenomas in the small intestine and colon, resulting in a 64‐day reduction in median survival. These adenomas showed higher cell proliferation, reduced apoptosis, and increased dysplasia. Additionally, loss of 14‐3‐3*σ* promoted a mesenchymal‐like gene expression pattern in the tumors, similar to profiles observed in CRC associated with poor relapse‐free survival. The 14‐3‐3*σ* knockout was followed by overexpression of proteins contributing to tumor growth and progression, such as YAP1, SNAIL, c‐JUN, and FOXO1. Overall, the study suggested 14‐3‐3*σ* as a tumor suppressor in the intestine whose expression level is negatively linked to metastasis and worse outcomes in patients suffering CRC [53]. In concordance with this study, Liu et al. reported 14‐3‐3*σ* as a tumor suppressor in CRC. Indeed, a hypoxic microenvironment promotes tumorigenesis and tumor progression in various cancers, including CRC. To overcome the hypoxic condition, tumor cells activate various hypoxia‐responsive signaling pathways, many of which are regulated by hypoxia‐inducible factor‐1 (HIF‐1), a transcription factor that activates genes involved in metabolic adaptation, tumor growth, and angiogenesis. Liu et al. indicated that 14‐3‐3*σ* negatively regulates hypoxia‐induced CRC metastasis and angiogenesis by binding to HIF‐1*α* and decreasing its stability. 14‐3‐3*σ* causes HIF‐1*α* ubiquitination, followed by proteasome‐mediated degradation by implicating the E3 ligase NEDD4L. Bevacizumab and 14‐3‐3*σ* combined delivery in xenograft models of CRC was accompanied by repression of tumor growth. Thereby, the study suggested the 14‐3‐3*σ*/NEDD4L/HIF‐1*α* axis as a potential therapeutic target for CRC [54]. LASP1, an upregulated protein in various cancer types, contributes to CRC progression by stimulation of the PI3K/AKT signaling pathway through the regulation of the AKT phosphorylation level. Shao et al. revealed that this effect is mediated by 14‐3‐3*σ* contribution (Figure 3). It attaches to AKT and prevents LASP1‐dependent activation of AKT kinase in CRC. In addition, the study supported the issue that LASP1‐14‐3‐3*σ* interaction reduces the stability of 14‐3‐3*σ*, inhibiting its effects on the PI3K/AKT pathway. 14‐3‐3*σ* can impact the migration and invasion potential of CRC cells. Transfection of SW480 and HCT116 cell lines with siRNA targeting 14‐3‐3*σ* led to improved migratory capacity. Conversely, the induction of 14‐3‐3*σ* in SW‐480 cells reduced cell migration, invasion, and motility. So, 14‐3‐3*σ* is suggested as a potential therapeutic target for AKT‐activated cancers [55].

14‐3‐3 proteins could cause drug resistance in CRC. 14‐3‐3*σ* acts as an oncogene in CRC and is associated with drug resistance. In the absence of 14‐3‐3*σ*, transcription factor Yin Yang1 (YY1) binds to the promoter region of the genes involved in the unfolded protein response (UPR) pathway, leading to decreased gene expression. 14‐3‐3*σ* interacts with YY1, causing it to transfer to the cytoplasm, preventing its inhibitory effect on the expression of UPR pathway genes. Lonare et al. showed that 14‐3‐3*σ* contributes to the drug resistance of CRC, and knockdown of this member of the 14‐3‐3 family is followed by induced sensitivity to 5‐fluorouracil (5FU) [68].

Epigenetic changes are pivotal regulators of cancer development. These changes can occur by dysregulation of histone deacetylases (HDACs) and the alterations in acetylation content of histone and nonhistone proteins. Implying HDAC inhibitors help in reversing aberrant acetylation patterns in cancers. The function of sulforaphane (SFN), a well‐known HDAC inhibitor in human colon cancer cells, is mediated by the regulation of 14‐3‐3 proteins. Indeed, SFN causes 14‐3‐3 to accumulate in the nucleus and binds to HDAC3, inactivating its NLS, causing its translocation to the cytosol [69].

**Figure 4:**
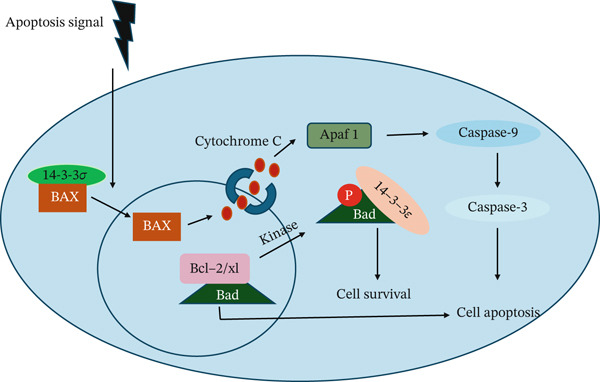
Regulation of cell death by 14‐3‐3 proteins. Bad binds to antiapoptotic factor bcl‐2/xl in the mitochondria and reverses their death repressor activity_._ 14‐3‐3*ε* binds to phosphorylated Bad, preventing its import to the mitochondria, causing cell survival. 14‐3‐3*σ* binds to BAX, preventing its importation to the mitochondria. Apoptosis signals, such as DNA damage, dissociate 14‐3‐3*σ* from BAX, causing BAX to enter the mitochondria and stimulate cytochrome c release from mitochondria and trigger the caspase cascade and apoptosis.

14‐3‐3 proteins could be a target for nonsteroidal anti‐inflammatory drugs that induce apoptosis. Sulindac sulfide inhibits the promoter of 14‐3‐3*ε*, preventing its transcription. 14‐3‐3*ε* binds to phosphorylated Bad in the cytosol, preventing it from translocating to the mitochondria and triggering apoptosis. However, apoptotic signals cause Bad to detach from 14‐3‐3*ε* and transfer to the mitochondria, thereby stimulating apoptosis through the mitochondrial pathway (Figure 4) [56].

In contrast to the previous study that described 14‐3‐3*ε* as an oncogene in CRC, Guo et al. reported 14‐3‐3*ε* as an antitumor protein during the investigation of the underlying mechanism of the tumor suppressor effects of triptolide (TP), a natural compound that inhibits the proliferation and invasion of CRC cells. Their findings showed that 14‐3‐3*ε* causes hnRNP C export to the cytoplasm, preventing its functioning in the nucleus, which was followed by inhibited proliferation, cell‐cycle arrest at the S phase, and autophagy in SW480 cells treated with TP. Hence, 14‐3‐3*ε* plays a significant role in TP‐mediated regulation of cell cycle, proliferation, and autophagy in CRC. [57, 70].

14‐3‐3*ε* proteins have a prognostic value in CRC. It has been indicated that 14‐3‐3*ε* localizes to the nucleus, and its expression is associated with lymph node metastasis in patients with CRC. Although no significant association was found between cytosolic 14‐3‐3*ε* expression and clinicopathological features, nuclear 14‐3‐3*ε* expression is suggested to be a valuable prognostic biomarker, as a reduction in its nuclear expression is an indicator of worsening clinical outcomes in CRC patients [58].

14‐3‐3*ζ* serves as a crucial protein in numerous signal transduction pathways and is critically involved in tumor development. Growing evidence has indicated overexpression of 14‐3‐3*ζ* across various cancer types, functioning as an oncogene, and influencing a broad spectrum of cellular processes such as cell cycle regulation, proliferation, migration, invasion, and apoptosis [71]. Li Y et al. found overexpression of 14‐3‐3*ζ* in both mRNA and protein levels in 46 CRC tissues by qRT‐PCR and IHC [61]. Metformin, a well‐known drug for diabetes mellitus, has been recently suggested to improve CRC outcomes. Ding et al. investigated whether metformin could exert its anticancer effects on CRC through the regulation of 14‐3‐3*ζ*. They observed increased expression of 14‐3‐3*ζ* in CRC tissues compared with adjacent normal tissues, as well as in CRC tissues from patients with diabetes compared with those without diabetes. Additionally, their findings revealed that CRC cell lines with higher 14‐3‐3*ζ* expression (HCT115 and SW480) responded better to metformin compared with cell lines with lower 14‐3‐3*ζ* expression (HCT116 and RKO). Knockdown of 14‐3‐3*ζ* in SW480 and HCT15 cells led to suppressed cell proliferation, slowed tumor growth, and enhanced apoptosis. Inhibition of 14‐3‐3*ζ* caused higher amounts of phosphorylated AMPK and decreased levels of phosphorylated mTOR. Therefore, 14‐3‐3*ζ*, negatively regulated by metformin, is considered a tumor–promoting factor in CRC acting by the regulation of the AMPK‐mTOR pathway [59]. A key mitosis regulator, thyroid hormone Receptor Interactor 13 (TRIP13), is overexpressed in CRC, stimulating cell proliferation, migration, and invasion. Sheng et al., exploring the underlying mechanism of these events, found 14‐3‐3*ζ* as a protein binding to TRIP13, mediating G2‐M transition and EMT. Knockdown of 14‐3‐3*ζ* led to repression of migration and invasion capability in both HCT116 and SW480 cells [60]. miR‐451 has been found to be a tumor suppressor miRNA, downregulated in CRC tissues. This miRNA targets and inhibits 14‐3‐3*ζ*, preventing its binding to FOXO3, an inhibitory transcription factor contributing to the inhibition of cancer progression. This causes the nuclear accumulation of FOXO3 and inhibition of the expression of genes involved in proliferation and viability of CRC cells [61].

14‐3‐3*β* acts as an oncogene in certain cancers and is found upregulated in various types of cancer. O′Dwyer et al., following proteomics analysis, found 14‐3‐3*β* as an upregulated protein in CRC with a significant association with microsatellite instability. Tumors lacking 14‐3‐3*β* immunoreactivity were correlated with a better prognosis, with mean survival times of 129 months in patients negative for 14‐3‐3*β* and 107 months in those showing positive expression [62]. Also, a multivariate analysis in patients with CRC conducted by Ahluwalia et al. found a high prognostic score for 14‐3‐3*β* to be considered as an informative biomarker of poor prognosis [72]. 14‐3‐3*β* could exert its oncogenic roles in CRC by regulating the PI3K/AKT signaling pathway. According to Zhou et al., the protein levels of p‐PI3K and p‐AKT were reduced after 14‐3‐3*β* knockdown, while overexpressing PIK3R2 reversed these events, suggesting that 14‐3‐3*β* can interact with PIK3R2, a PI3K p85 subunit family member, positively regulating its expression. This event causes upregulation of PI3K‐AKT signaling. Inhibition of 14‐3‐3*β* reduced proliferation, induced cell‐cycle arrest in G0/G1, and elevated apoptosis in CRC cells [63].

Although the oncogenic role of 14‐3‐3*γ* and its contribution to the metastasis process were reported by Lee et al. [73] and others, less was known about the specific role of this member of the 14‐3‐3 family in CRC. In 2024, Wang et al. reported 14‐3‐3*γ* as the most markedly overexpressed member of the 14‐3‐3 family in CRC tissues, associating with a poor prognosis. In addition, their data indicated that 14‐3‐3*γ* stimulated the CRC cells′ proliferation, migration, and invasion. Furthermore, RNA‐seq data supported the involvement of Wnt and EMT pathways in the regulation of CRC by 14‐3‐3*γ*. *β*‐catenin was found to be a binding protein for 14‐3‐3*γ*, mediating its oncogenic activities by stimulating the Wnt/*β*‐catenin signaling in CRC cells. Thereby, the study suggested 14‐3‐3*γ* as a promising therapeutic target for the treatment of CRC [64].

Immune checkpoint blockade (ICB) has revolutionized CRC treatment, particularly in cancers with a wide range of mutations, such as CRC. Immune checkpoints become active when proteins on the surface of T cells detect and attach to specific proteins on other cells, such as certain tumor cells. These proteins are referred to as immune checkpoint proteins. When the checkpoint and partner proteins connect, they transmit an “off” signal to the T cells, which can inhibit the immune system′s ability to attack the cancer. Immune checkpoint inhibitors (ICIs) are immunotherapeutic agents that prevent checkpoint proteins from binding to their partner proteins. This blocks the “off” signal, enabling T cells to effectively target and destroy cancer cells [74, 75]. Despite tremendous advances in clinical therapy, many patients remain resistant to ICB therapy. Furthermore, patients who initially responded favorably often later experienced disease recurrence and progression. Li et al., exploring the underlying mechanism of this resistance, conducted an immunohistochemical analysis on 20 pairs of primary CRC and paracancerous tissues. They found significant upregulation of 14‐3‐3*η* in CRC tissues compared with paracancerous tissues and a stimulating role of 14‐3‐3*η* on the growth and movement of CRC cells. In addition, they assessed the association between 14‐3‐3*η* and immune markers of CRC patients and uncovered a significant correlation between 14‐3‐3*η* expression levels and microsatellite instability, interferon gamma scores, Merck18, and programmed death‐ligand 1 (PD‐L1) scores. In addition, their findings revealed a notable positive relationship between 14‐3‐3*η* and immune checkpoints such as PDCD1, CTLA4, LAG3, CD274, HAVCR2 (TIM‐3), and TIGIT in CRC, supporting the idea that 14‐3‐3*η* may contribute to tumor immune evasion during the development of the tumor. Increased expression of 14‐3‐3*η* was associated with reduced sensitivity to ICIs and elevated levels of CD8+ T cell exhaustion markers, suggesting a potential mechanism of immunotherapy resistance in CRC patients led by 14‐3‐3*η*‐induced exhaustion in CD8+ T cells [65].

Although the molecular mechanisms of the oncogenic roles of 14‐3‐3*τ* are well investigated in breast cancer, there is no experimental research focusing on the contribution of 14‐3‐3*τ* in CRC. However, during a proteomic analysis conducted by Larriba et al., 14‐3‐3*τ* was found, along with other 14‐3‐3 isoforms *β*, *ε*, *σ*, and *ζ*, downregulated in HT29 cells after Snail1 expression, a transcription factor involved in the EMT process [76].

Although numerous studies have shed light on the functions of 14‐3‐3 proteins in CRC, there is a gap in the molecular mechanisms by which 14‐3‐3 proteins could regulate CRC. In addition to the limited number of studies focusing on this issue and the scarcity of valuable information about isoform tau, contradictory results exist, particularly regarding 14‐3‐3*σ*, which has been reported both as a tumor suppressor and as an oncogenic factor, depending on the experimental context. Such discrepancies highlight the need for standardized methodologies, larger patient cohorts, and more rigorous clinical validation. Third, most studies focus on single isoforms, whereas the interplay between different isoforms and their combined effects on CRC biology remains largely unexplored. Finally, limited data are available on the long‐term clinical implications of targeting 14‐3‐3 proteins, making translational application still challenging at this stage.

## 5. Context‐Dependent Roles of 14‐3‐3 Proteins in CRC

Accumulating evidence indicates that the functional roles of 14‐3‐3 isoforms in CRC are highly context‐dependent rather than intrinsically oncogenic or tumor suppressive. Several factors may account for the contradictory findings reported across studies.

First, tumor stage appears to be a critical determinant. Certain isoforms, such as 14‐3‐3*σ*, exhibit tumor‐suppressive functions in early‐stage CRC by inducing cell‐cycle arrest and limiting genomic instability, whereas in advanced tumors they may promote survival, therapy resistance, or metastasis [53, 68].

Second, subcellular localization strongly influences functional outcomes. Compared with the cytoplasmic localization of 14‐3‐3 proteins, their nuclear distribution increases their accessibility to transcription factors, kinases, and apoptotic regulators, thereby impacting their biological effects. For instance, nuclear localization of specific isoforms has been linked to improved prognosis, whereas cytoplasmic accumulation often correlates with tumor progression [23, 38, 58].

Third, isoform‐specific interacting partners define downstream signaling consequences. Binding to oncogenic pathways such as PI3K/AKT or Wnt/*β*‐catenin promotes tumor progression, whereas interactions with tumor suppressive proteins such as FOXO or HIF‐1*α* regulators may inhibit CRC growth and metastasis [54, 55, 64].

Finally, microenvironmental factors, including hypoxia and immune context, further modulate 14‐3‐3 function, contributing to immune evasion and therapy resistance [54, 65]. Collectively, these variables explain the apparent discrepancies in the literature and underscore the necessity of interpreting 14‐3‐3 function within a multidimensional biological framework.

## 6. Clinical Implications of 14‐3‐3 Proteins in CRC

Growing evidence reveals that, beyond their established biological activities, 14‐3‐3 proteins hold significant clinical value in the diagnosis, prognosis, and therapeutic strategies for CRC.

Isoform‐specific dysregulation of 14‐3‐3 proteins distinguishes CRC tissues from adjacent normal mucosa. Differential expression patterns of 14‐3‐3 isoforms suggest their potential utility as molecular biomarkers. For example, Young et al. observed a 1.3‐fold and a 4.4‐fold decrease in the expression of 14‐3‐3*η* and *σ*, respectively, in CRC tissues compared with adjacent normal tissues. The elevated expression of oncogenic isoforms, such as 14‐3‐3*ζ*, *β*, and *γ*, correlates with advanced tumor stage, lymph node metastasis, epithelial–mesenchymal transition, and reduced overall survival [59, 62, 64]. In contrast, the loss of tumor‐suppressive isoforms, particularly 14‐3‐3*σ*, is associated with aggressive tumor behavior and unfavorable clinical outcomes [53]. These data support the potential of 14‐3‐3 proteins as prognostic biomarkers in CRC, although none have yet been clinically validated as standalone biomarkers. Importantly, subcellular localization further refines prognostic value, as the nuclear versus cytoplasmic distribution of specific isoforms has been linked to distinct patient survival profiles.

Beyond prognosis, 14‐3‐3 proteins exhibit predictive value for therapeutic response. Overexpression of certain isoforms, such as 14‐3‐3*σ*, contributes to resistance against chemotherapy agents such as 5FU and platinum‐based therapies via modulation of survival and DNA damage response pathways [68]. In addition, recent studies have revealed that overexpression of 14‐3‐3*η* causes immune checkpoint inhibitor resistance through CD8^+^ T‐cell exhaustion [65]. These findings suggest that assessing 14‐3‐3 expression may help stratify patients for personalized treatment strategies.

Therapeutically, targeting 14‐3‐3 proteins has emerged as a promising yet challenging strategy. Research into developing 14‐3‐3 inhibitors has been conducted to explore the biological roles of 14‐3‐3 proteins and to identify potential therapeutic strategies. Notably, peptide inhibitors like R18 and difopein have made considerable progress in advancing the understanding of 14‐3‐3 functions. Furthermore, there is a growing interest in small‐molecule inhibitors that target 14‐3‐3 protein–protein interactions. Small molecules that target 14‐3‐3 proteins have emerged as a promising therapeutic strategy, primarily through disruption of 14‐3‐3–client protein interactions. Early proof‐of‐concept studies identified compounds such as BV02 and related derivatives that interfere with the binding of 14‐3‐3 proteins to prosurvival partners, leading to apoptosis and inhibition of growth in cancer cells. For instance, BV02 was shown to disrupt 14‐3‐3*σ*–c‐Abl interactions, resulting in reactivation of apoptotic signaling pathways in tumor models [77]. In addition, structure‐guided approaches have yielded small molecules such as FOBISIN101 and other phosphopeptide‐mimetic compounds that selectively bind to the amphipathic groove of 14‐3‐3 proteins, thereby blocking their interaction with oncogenic clients [78]. Given the well‐documented oncogenic roles of several 14‐3‐3 isoforms in CRC progression and therapy resistance, pharmacological targeting of these proteins represents a compelling translational opportunity. Although direct evaluation of 14‐3‐3 inhibitors in CRC models remains limited, these inhibitors have demonstrated antiproliferative effects across multiple cancer cell lines, providing a strong rationale for future investigation of 14‐3‐3 inhibitors in CRC preclinical models.

More recently, attention has shifted toward exploiting 14‐3‐3 dependency in therapy‐resistant tumors. Preclinical studies suggest that pharmacological disruption of 14‐3‐3 interactions can sensitize cancer cells to chemotherapy and metabolic stress, supporting combination strategies rather than monotherapy [79]. Although most small‐molecule 14‐3‐3 inhibitors remain at the preclinical stage and lack isoform specificity, these studies collectively suggest the therapeutic potential of targeting 14‐3‐3 protein–protein interactions in CRC and related solid tumors.

Collectively, these clinical implications position 14‐3‐3 proteins as multifunctional biomarkers and potential therapeutic targets in CRC, supporting their integration into precision oncology frameworks. However, the dual and context‐dependent roles of 14‐3‐3 proteins underscore the necessity of isoform‐selective and context‐aware therapeutic approaches to minimize off‐target effects.

## 7. Conclusion and Future Perspectives

14‐3‐3 proteins represent a highly conserved family of regulatory molecules that orchestrate diverse biological processes through extensive protein–protein interactions, thereby influencing key signaling pathways involved in proliferation, migration, invasion, apoptosis, and stress responses. Accumulating evidence has indicated that 14‐3‐3 proteins play a role in cancer initiation and progression. They can function as either tumor suppressors or oncogenes in CRC. Dysregulation of 14‐3‐3 isoforms impairs crucial signaling pathways, including Wnt/*β*‐catenin, PI3K/AKT, HIF‐1*α*, and immune checkpoint regulation, thereby influencing CRC initiation, progression, metastasis, and therapeutic response (Table 3).

**Table 3:** Major signaling pathways regulated by 14‐3‐3 proteins in CRC.

Signaling pathway	Involved isoform(s)	Mechanism	Biological effect
PI3K/AKT	*β*/*σ*	Interaction with PIK3R2 stimulating PI3K/AKT pathway/binding to AKT and preventing LASP1‐dependent activation of AKT kinase	Stimulating proliferation and inhibits apoptosis/suppresses invasion and migration
Wnt/*β*‐catenin	*γ*	Interaction with *β*‐catenin	Promoting proliferation, invasion, and EMT
HIF‐1*α* signaling	*σ*	Promoting degradation of HIF‐1 via implication of NEDD4L	Suppressing angiogenesis and tumor growth
AMPK/mTOR	*ζ*	Inhibition of AMPK signaling	Promoting proliferation and inhibits apoptosis
Apoptosis	*ε*, *ζ*	Binding and inhibiting proapoptotic proteins	Cell survival
Immune checkpoint regulation	*η*	Increasing PD‐L1 and CD8+ T cell exhaustion	Immunotherapy resistance

The dual and sometimes contradictory roles of 14‐3‐3 proteins underscore the complexity of their biology in CRC. Rather than acting as fixed oncogenes or tumor suppressors, we propose a context‐dependent and isoform‐specific model in which the functional outcomes of 14‐3‐3 proteins are determined by tumor stage, subcellular localization, interacting partners, and microenvironmental cues. This framework helps reconcile inconsistent findings reported across studies and highlights the necessity of interpreting 14‐3‐3 function within a multidimensional biological context. Many investigations have concentrated on general functions of 14‐3‐3, whereas the molecular roles of individual isoforms are still poorly understood.

From a clinical perspective, differential expression and localization of specific 14‐3‐3 isoforms suggest potential diagnostic, prognostic, and predictive relevance in CRC; however, none has yet been clinically validated as standalone biomarkers. Translating these observations into clinical practice will require well‐designed translational studies, including large patient cohorts, standardized detection methods, and isoform‐resolved analyses. Another promising avenue is the therapeutic targeting of 14‐3‐3 proteins. Developing small molecules that either block or stabilize specific 14‐3‐3 isoform interactions could provide new approaches to control major signaling pathways, including PI3K/AKT, Wnt/*β*‐catenin, and HIF‐1*α*. Additionally, treatments that target 14‐3‐3 proteins may work better when combined with ICIs, chemotherapy, or radiation therapy. These combinations could improve patient outcomes by lowering resistance. However, in order to fully realize the clinical potential of 14‐3‐3 proteins in CRC, more translational research and a deeper mechanistic understanding will be required.

## Author Contributions

S.D. and A.S.M. contributed to investigating, data gathering, and writing the original draft of the manuscript; M.K., A.M.H., M.T.C., and S.F. contributed to writing the main text of the manuscript; Y.G.B. and S.B. revised and provided the final draft; S.J., H.G., and D.M, and Z.H. prepared the figures and tables; F.A.B. and Q.B. contributed to hypothesis, scientific, and structural editing, supervision, and verifying the manuscript before submission.

## Funding

No funding was received for this manuscript.

## Ethics Statement

The authors have nothing to report.

## Consent

The authors have nothing to report.

## Conflicts of Interest

The authors declare no conflicts of interest.

## Data Availability

Data sharing is not applicable to this article as no datasets were generated or analyzed during the current study.
